# A *Drosophila* model of combined D-2- and L-2-hydroxyglutaric aciduria reveals a mechanism linking mitochondrial citrate export with oncometabolite accumulation

**DOI:** 10.1242/dmm.035337

**Published:** 2018-09-21

**Authors:** Hongde Li, Alexander J. Hurlburt, Jason M. Tennessen

**Affiliations:** Department of Biology, Indiana University, 1001 East Third Street, Bloomington, IN 47405, USA

**Keywords:** 2-Hydroxyglutarate, Oncometabolite, L2HGDH, *Scheggia*, SLC25A1

## Abstract

The enantiomers of 2-hydroxyglutarate (2HG) are potent regulators of metabolism, chromatin modifications and cell fate decisions. Although these compounds are associated with tumor metabolism and commonly referred to as oncometabolites, both D- and L-2HG are also synthesized by healthy cells and likely serve endogenous functions. The metabolic mechanisms that control 2HG metabolism *in vivo* are poorly understood. One clue towards how cells regulate 2HG levels has emerged from an inborn error of metabolism known as combined D- and L-2HG aciduria (D-/L-2HGA), which results in elevated D- and L-2HG accumulation. Because this disorder is caused by mutations in the mitochondrial citrate transporter (CIC), citrate must somehow govern 2HG metabolism in healthy cells. The mechanism linking citrate and 2HG, however, remains unknown. Here, we use the fruit fly *Drosophila melanogaster* to elucidate a metabolic link between citrate transport and L-2HG accumulation. Our study reveals that the *Drosophila* gene *scheggia* (*sea*), which encodes the fly CIC homolog, dampens glycolytic flux and restricts L-2HG accumulation. Moreover, we find that *sea* mutants accumulate excess L-2HG owing to elevated lactate production, which inhibits L-2HG degradation by interfering with L-2HG dehydrogenase activity. This unexpected result demonstrates that citrate indirectly regulates L-2HG stability and reveals a feedback mechanism that coordinates L-2HG metabolism with glycolysis and the tricarboxylic acid cycle. Finally, our study also suggests a potential strategy for preventing L-2HG accumulation in human patients with CIC deficiency.

This article has an associated First Person interview with the first author of the paper.

## Abstract (precis)

**Summary:** This study reveals a mechanism that links export of mitochondrial citrate to accumulation of the oncometabolite L-2-hydroxyglutarate, suggesting a potential treatment for individuals with combined D-2- and L-2-hydroxyglutaric aciduria, a rare inborn error of metabolism.

## INTRODUCTION

Although the enantiomers of 2-hydroxyglutarate (2HG) have emerged as potent oncometabolites capable of influencing a wide range of cellular processes, both D- and L-2HG are also produced by healthy tissues (Ye et al., 2018). Mammalian cells produce D-2HG as a result of γ-hydroxybutyrate metabolism and phosphoglycerate dehydrogenase activity (Fan et al., 2015; Struys et al., 2005b), whereas L-2HG is generated by malate dehydrogenase and lactate dehydrogenase A in response to hypoxia, acidic cellular conditions and decreased electron transport chain activity (Intlekofer et al., 2015, 2017; Oldham et al., 2015; Reinecke et al., 2012; Nadtochiy et al., 2016; Mullen et al., 2011; Teng et al., 2016). Furthermore, yeast and *Drosophila* generate D- and L-2HG, respectively, under standard growth conditions (Becker-Kettern et al., 2016; Li et al., 2017). Overall, these results suggest that D- and L-2HG serve endogenous biological functions and emphasize the need to understand how 2HG metabolism is controlled *in vivo*.

Despite the fact that D- and L-2HG can dramatically influence the cellular physiology of healthy tissues, most 2HG studies focus on the role of these compounds in cancer cell lines and, as a result, the molecular mechanisms that regulate endogenous 2HG accumulation remain poorly understood. In fact, most of our current understanding about endogenous D- and L-2HG metabolism stems from a class of rare human diseases that are collectively known as the 2HG acidurias (2HGAs) (Kranendijk et al., 2012). For example, patients with L2HGA accumulate L-2HG owing to loss-of-function mutations in the FAD-dependent enzyme L-2HG dehydrogenase (L2HGDH) (Rzem et al., 2004), which converts L-2HG to 2-oxoglutarate (2OG). Similarly, D2HGA type I results from the absence of D-2HG dehydrogenase (D2HGDH) activity and an inability to degrade D-2HG (Struys et al., 2005a). Overall, these studies illustrate how the 2HGA disorders provide essential clues for understanding endogenous 2HG metabolism.

In addition to the disorders associated with a single 2HG enantiomer, a small subset of 2HGA patients exhibit elevated levels of both D- and L-2HG. This rare disease, which is known as combined D-/L-2HGA, results in severe neurological and muscular defects, developmental delays and childhood lethality (Muntau et al., 2000). Considering that this disorder is caused by loss-of-function mutations in the mitochondrial citrate carrier (CIC; encoded by *SLC25A1*) (Nota et al., 2013; Palmieri, 2013), citrate transport must somehow govern 2HG accumulation. However, the mechanism linking these metabolites is unknown.

We recently discovered that *Drosophila* larvae accumulate high concentrations of L-2HG during normal larval growth (Li et al., 2017). Moreover, we determined that flies, like mammals, rely on lactate dehydrogenase (dLDH; FBgn0001258) to synthesize L-2HG from the tricarboxylic acid (TCA) cycle intermediate 2OG (Li et al., 2017). These findings demonstrate that fundamental aspects of L-2HG metabolism are conserved between flies and humans, and suggest that studies in *Drosophila* will be essential for understanding how L-2HG accumulation is controlled *in vivo*. Here, we exploit the fly model to examine the metabolic link between citrate and L-2HG. By studying a hypomorphic mutation in the *Drosophila* gene *scheggia* (*sea*; FBgn0037912), which encodes the fly *SLC25A1* homolog (Carrisi et al., 2008; Morciano et al., 2009), we demonstrate that loss of mitochondrial citrate efflux results in elevated glucose catabolism, increased lactate production and enhanced L-2HG accumulation. The elevated L-2HG levels observed in *sea* mutants, however, are not the result of excess synthesis, but rather are caused by decreased degradation. Moreover, our studies indicate that *sea* mutants accumulate excess L-2HG as a result of increased lactate synthesis, which inhibits the enzyme that degrades L-2HG, dL2HGDH (FBgn0032729) (Li et al., 2017). Overall, our findings present a metabolic feedback loop by which L-2HG levels are controlled by the combined outputs of glycolysis and the TCA cycle, and suggest that a similar mechanism could be active in mammals.

## RESULTS

### *sea* mutant larvae accumulate excess L-2HG

**Fig. 1.**
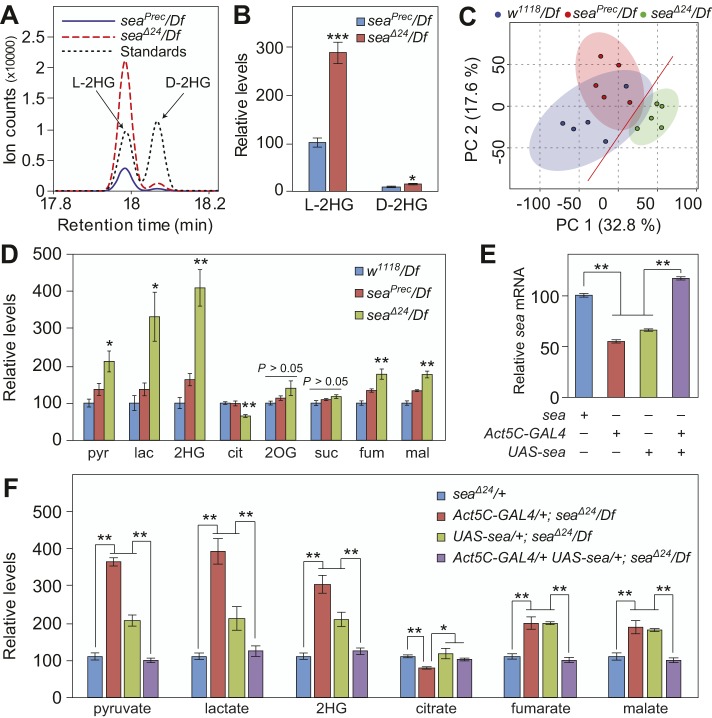
***sea* mutant larvae accumulate excess L-2HG.** (A) L- and D-2HG in larvae were detected separately using a chiral derivatization method coupled with GC-MS. (B) Relative abundance of L-2HG and D-2HG in *sea* mutant (*sea*^Δ*24*^*/Df*) and control (*sea^Prec^/Df*) larvae. (C) The PCA score plots of GC-MS spectra show that the metabolic profile of *sea*^Δ*24*^*/Df* mutants is significantly different from that of the *sea^P^^rec^/Df* and *w^1118^/Df* controls. (D) Targeted GC-MS analysis reveals that *sea*^Δ*24*^*/Df* mutants display significant changes in pyruvate (pyr), lactate (lac), 2-hydroxyglutarate (2HG), citrate (cit), fumarate (fum) and malate (mal). 2-oxoglutarate (2OG) and succinate (suc) were not significantly altered in the mutant. (E,F) Ubiquitous expression of a *UAS-sea* transgene restores *sea* mRNA levels (E) in *sea* mutant larvae and rescues the metabolic phenotypes (F). For all panels, data are shown as mean±s.e.m., *n*=5 samples containing 15 mid-L3 larvae collected from independent mating bottles. *P<0.05, **P<0.01. Data were analyzed using a two-tailed Student's *t*-test with Bonferroni correction for multiple comparisons. Data are representative of at least two independent experiments.

To determine whether the *Drosophila* homolog of *SLC25A1* influences 2HG accumulation, we used gas chromatography-mass spectrometry (GC-MS) to quantify both D- and L-2HG in *sea* mutant larvae (*sea*^Δ*24*^*/Df*), which exhibit a significant reduction in mitochondrial CIC activity (Morciano et al., 2009). When compared with a genetically matched control strain (*sea^Prec^/Df*), both D- and L-2HG were significantly elevated in *sea* mutants (Fig. 1A,B), with L-2HG representing the majority of the 2HG pool. Although these observations differ from patients with combined D-/L-2HGA, in which D-2HG is the more abundant enantiomer (Muntau et al., 2000), the metabolic profile of *sea*^Δ*24*^*/Df* mutants clearly indicates that the inverse relationship between CIC activity and L-2HG accumulation is present in flies.

### Glycolysis and the TCA cycle are disrupted in *sea* mutants

Combined D-/L-2HGA patients not only exhibit increased 2HG levels and decreased citrate accumulation, but also possess elevated levels of lactate, 2OG, succinate, fumarate and malate (Nota et al., 2013; Prasun et al., 2015). To determine whether *sea* mutants display similar metabolic defects, we used GC-MS-based metabolomics to quantify the relative abundance of metabolites in glycolysis and the TCA cycle. Multivariate analysis of the resulting data sets revealed that *sea*^Δ*24*^*/Df* mutant larvae exhibit a distinct metabolic profile when compared with either *sea^Prec^/Df* or *w^1118^/Df* controls (Fig. 1C). Targeted analysis of these data revealed that *sea*^Δ*24*^*/Df* mutants and combined D-/L-2HGA patients display similar metabolic phenotypes, including decreased citrate levels and elevated concentrations of pyruvate, lactate, fumarate and malate (Fig. 1D). Similar metabolic changes were observed when the *sea*^Δ*24*^ mutation was analyzed in a second genetic background (in trans to a second deficiency that also uncovers the *sea* locus; Fig. S1). Moreover, the *sea*^Δ*24*^*/Df* metabolic phenotypes were rescued by ubiquitous expression of a *sea* complementary DNA (cDNA) from a *UAS-sea* transgene, indicating that the metabolic profile displayed by *sea*^Δ*24*^*/Df* mutants specifically results from the loss of CIC activity (Fig. 1E,F).

### Glycolytic flux is elevated in *sea* mutants

**Fig. 2.**
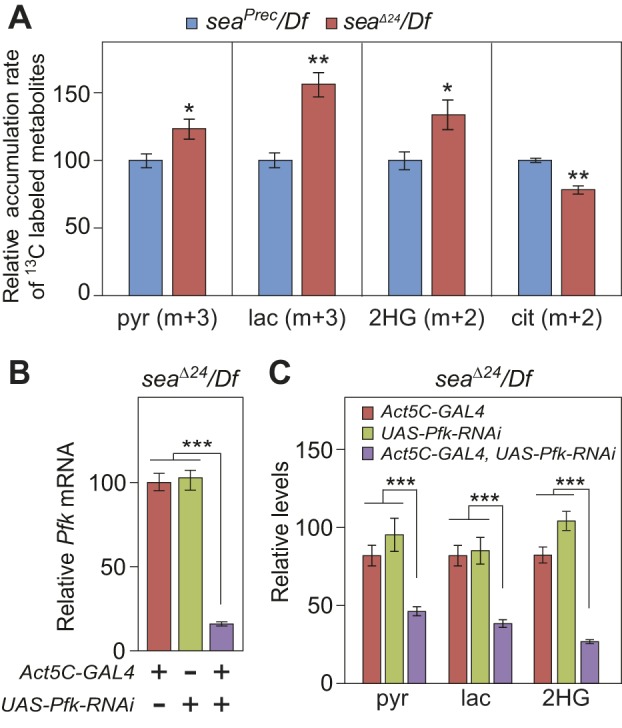
***sea* mutants exhibit elevated levels of glycolytic flux.** (A) The relative metabolic flux rates from ^13^C_6_-glucose into pyruvate (pyr), lactate (lac), 2HG and citrate (cit). *n*=4. (B) Relative *Pfk* mRNA levels in *sea*^Δ*24*^*/Df* mutant larvae that ubiquitously express a *UAS-Pfk-RNAi* transgene. *n*=3 samples containing 15 mid-L3 larvae collected from independent mating bottles. (C) *Pfk-RNAi* reduces pyruvate, lactate, and 2HG levels in *sea* mutant larvae. *n*=6 samples containing 15 mid-L3 larvae collected from independent mating bottles. All data are shown as mean±s.e.m. *P<0.05, **P<0.01. ***P<0.001. Data were analyzed using a two-tailed Student's *t*-test with Bonferroni correction for multiple comparisons. Data are representative of at least two independent experiments.

Considering that *Drosophila* larvae primarily synthesize pyruvate, lactate and L-2HG from glucose (Li et al., 2017; Tennessen et al., 2011), our data suggested that the *sea*^Δ*24*^*/Df* mutants accumulate excess L-2HG owing to increased glycolytic flux. We tested this hypothesis by feeding ^13^C_6_-glucose to both *sea*^Δ*24*^*/Df* mutants and *sea^P^^rec^/Df* controls, and selectively monitoring ^13^C incorporation into lactate, pyruvate, citrate and 2HG. When compared with the control strain, *sea*^Δ*24*^*/Df* larvae exhibited a 60% increase in the rate of lactate (m+3) synthesis, a modest increase in the accumulation rate of labeled pyruvate (m+3) and 2HG (m+2), and a slight, but significant, decrease in the rate of m+2 citrate synthesis (Fig. 2A). These observations confirm that glycolytic flux is elevated in *sea* mutants, and are consistent with a recent study that observed enhanced glucose consumption and increased lactate production in CIC-deficient human cells (Jiang et al., 2017).

To determine whether increased glycolytic flux is necessary for elevated L-2HG accumulation, we used a *Phosphofructokinase* (*Pfk*) RNAi transgene (*UAS-Pfk-RNAi*; FBgn0003071) to attenuate glycolysis in both control and mutant larvae. Ubiquitous expression of this construct in a wild-type background reduced *Pfk* mRNA levels by 80%, and induced a similar reduction in pyruvate, lactate and 2HG levels (Fig. S2A,B). Similarly, *Pfk-RNAi* expression in a *sea*^Δ*24*^*/Df* mutant background induced an 80% decrease in *Pfk* mRNA levels, a 75% decrease in 2HG and a ∼50% decrease in pyruvate and lactate (Fig. 2B,C). Overall, these results support a model in which *Drosophila* CIC activity restricts L-2HG accumulation by inhibiting glucose catabolism.

**Fig. 3.**
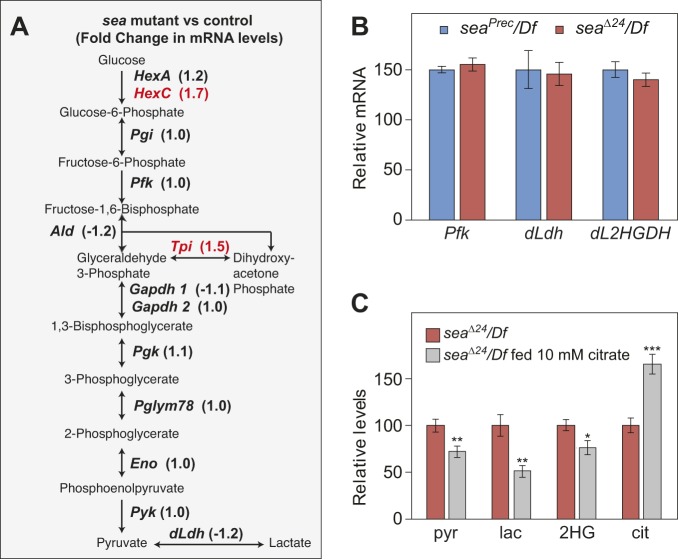
***Drosophila* CIC activity regulates glycolysis at a post-transcriptional level.** (A) A schematic diagram illustrating the *Drosophila* glycolytic pathway. The number in parentheses represents the change in gene expression observed in *sea* mutants. (B) A comparison of *Pfk*, *dLdh* and *dL2HGDH* mRNA levels in the *sea*^Δ*24*^*/Df* mutants and *sea^Prec^/Df* controls. *n*=3 samples containing 15 mid-L3 larvae collected from independent mating bottles. (C) *sea*^Δ*24*^*/Df* mutant larvae fed a semi-defined diet supplemented with 10 mM citrate for 24 h accumulated excess citrate (cit) and displayed significant decreases in pyruvate (pyr), lactate (lac) and 2HG. *n*=6 samples containing 15 mid-L3 larvae collected from independent mating bottles. All data are shown as mean±s.e.m. *P<0.05, **P<0.01, ***P<0.001. Data were analyzed using a two-tailed Student's *t*-test. Data are representative of at least two independent experiments.

In order to further explore the mechanism linking citrate with glycolysis and L-2HG, we used RNA sequencing (RNA-seq) to determine whether *Drosophila* CIC activity influences the expression of glycolytic enzymes (Table S1). This analysis revealed that only two of the 25 *Drosophila* genes that encode glycolytic enzymes exhibited a >1.5-fold increase in *sea*^Δ*24*^*/Df* mutants, and no gene in this 25-gene subset exhibited a greater than twofold increase (Fig. 3A; Table S2). Quantitative reverse transcription PCR (qRT-PCR) analysis also confirmed that *Pfk*, *dLdh* and *dL2HGDH* mRNA levels were comparable between *sea*^Δ*24*^*/Df* mutants and *sea^P^^rec^/Df* controls (Fig. 3B). Moreover, we observed no changes in either *Malate dehydrogenase 1* or *2* gene expression (FBgn0262782; FBgn0262559; Table S1), both of which have been implicated in mammalian L-2HG synthesis (Ye et al., 2018). Overall, these results demonstrate that *Drosophila* CIC activity does not significantly influence the transcription of key genes directly associated with L-2HG metabolism and glycolysis.

Because our gene expression studies demonstrated that *Drosophila* CIC activity controls glycolysis at post-transcriptional levels, we next examined the possibility that changes in intracellular citrate distribution directly influence glycolysis and L-2HG metabolism. Considering that citrate inhibits glycolytic flux in mammals (Kemp and Gunasekera, 2002), and that cytosolic citrate is significantly depleted in *sea* mutants (Morciano et al., 2009), we tested whether exogenous citrate treatment could rescue the *sea* mutant phenotypes. Indeed, *sea*^Δ*24*^*/Df* mutants fed a citrate-supplemented diet for 24 h not only accumulated excess citrate but also exhibited a significant decrease in pyruvate, lactate and 2HG (Fig. 3C). These results indicate that citrate restricts glycolytic flux in *Drosophila* larvae and suggest that *sea* mutants accumulate excess L-2HG in response to decreased cytosolic citrate levels. Moreover, our observations support the findings of a recent human case study, in which a patient with combined D-/L-2HGA exhibited decreased urinary 2HG levels and reduced cardiac symptoms following citrate treatment (Muhlhausen et al., 2014).

### *sea* mutants accumulate excess L-2HG owing to decreased degradation

**Fig. 4.**
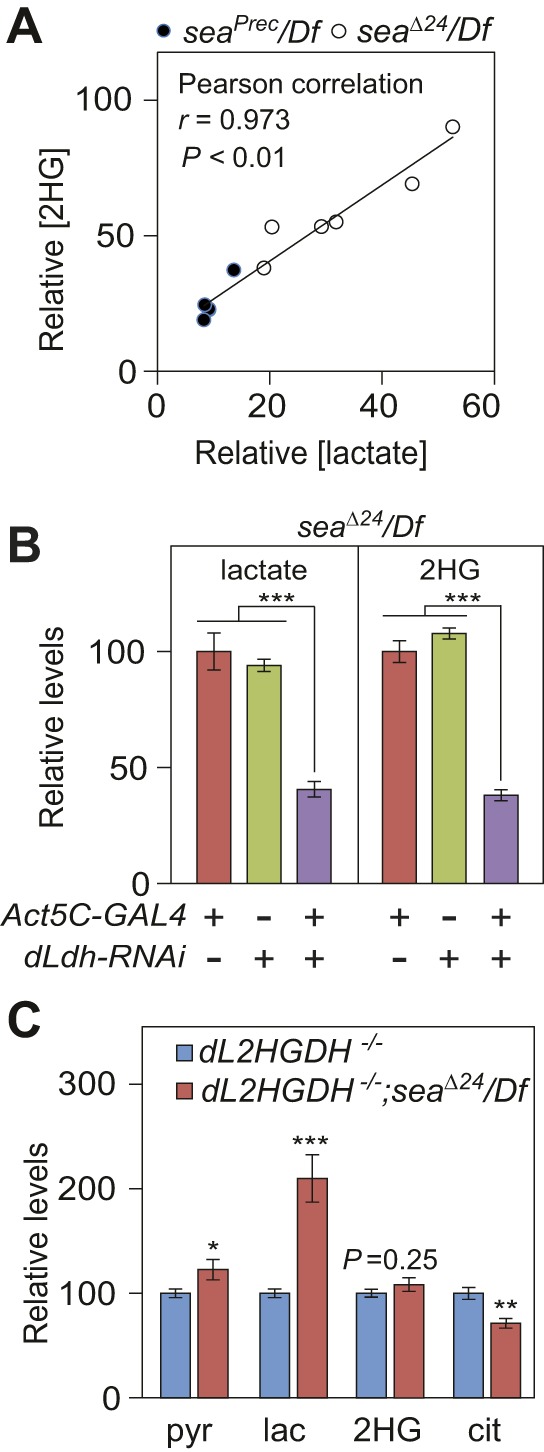
***sea* mutants accumulate excess L-2HG as a result of decreased degradation.** (A) GC-MS analysis of *sea*^Δ*24*^*/Df* mutants and *sea^Prec^/Df* controls reveals that lactate and 2HG levels are highly correlated in individual larval samples. (B) The relative abundance of lactate and 2HG in *sea*^Δ*24*^*/Df* mutant larvae that ubiquitously express a *UAS-dLdh-RNAi* (*dLdh*-RNAi) transgene. (C) The relative abundance of pyruvate (pyr), lactate (lac), 2HG and citrate (cit) in *dL2HGDH^12/14^* single mutants compared with *dL2HGDH^12/14^; sea*^Δ*24*^*/Df* double mutants. Note that 2HG levels are similar in both strains. Data are shown as mean±s.e.m., *n*=6 samples containing 15 mid-L3 larvae collected from independent mating bottles. *P<0.05, **P<0.01, ***P<0.001. Data were analyzed using a two-tailed Student's *t*-test with Bonferroni correction for multiple comparisons. Data are representative of at least two independent experiments.

The correlation between excess L-2HG accumulation and increased glycolytic flux does not necessarily indicate that *sea* mutants synthesize more L-2HG. Rather, elevated L-2HG levels could result from increased synthesis, decreased degradation or a combination of both processes. In this regard, we previously demonstrated that L-2HG synthesis and degradation are both regulated by lactate metabolism. Not only does *Drosophila* LDH directly synthesize L-2HG from 2OG, but lactate also stabilizes the larval L-2HG pool by inhibiting dL2HGDH activity (Li et al., 2017). Consistent with these earlier observations, we found a highly positive correlation between lactate and 2HG levels in both *sea*^Δ*24*^*/Df* mutants and *sea^P^^rec^/Df* controls (*r*=0.973, *P*<0.01; Fig. 4A), suggesting that *sea* mutants, similar to wild-type larvae, coordinate L-2HG accumulation with lactate production. We tested this possibility by measuring 2HG levels in *sea*^Δ*24*^*/Df* mutants that expressed a *UAS*-*dLdh-RNAi* transgene. Relative to the control strains, *dLdh-RNAi* depleted *dLdh* mRNA levels in *sea* mutants by 80% and induced a ∼60% reduction in both the lactate and 2HG pools (Fig. 4B; Fig. S3), demonstrating that dLDH influences L-2HG levels in mutant larvae.

The manner by which *sea* mutants accumulate L-2HG suggests two possible mechanisms: (1) *sea* mutants rely on dLDH to synthesize L-2HG at a higher rate than control larvae; (2) elevated lactate levels inhibit dL2HGDH activity and interfere with L-2HG degradation. We distinguished between these possibilities by measuring 2HG abundance in *dL2HGDH^12/14^; sea*^Δ*24*^*/Df* double mutants, which are able to synthesize, but not degrade, L-2HG. If loss of *Drosophila* CIC activity induces excess L-2HG synthesis, then *dL2HGDH^12/14^; sea*^Δ*24*^*/Df* double mutants should accumulate more L-2HG than the *dL2HGDH^12/14^* single mutant. In contrast, if *sea* mutants accumulate L-2HG owing to decreased degradation, then L-2HG levels will be similar in both genetic backgrounds. Our GC-MS analysis supports the latter model, as 2HG levels in *dL2HGDH; sea* double mutants were similar to those observed in *dL2HGDH; sea^P^^rec^* controls (Fig. 4C). In contrast, *dL2HGDH^12/14^; sea*^Δ*24*^*/Df* double mutants exhibited increased levels of lactate and pyruvate, as well as decreased citrate levels (Fig. 4C), suggesting that dL2HGDH does not influence these aspects of the *sea* mutant phenotype. This observation is also consistent with our findings that *dLdh* gene expression is normal in *sea*^Δ*24*^*/Df* larvae and that the L-2HG precursor, 2OG, is present at similar levels in both *sea* mutants and two control strains (Figs 1D and 3A). Overall, our studies suggest that *sea* mutants accumulate L-2HG as a result of lactate-dependent dL2HGDH inhibition and hint at a metabolic mechanism by which the L-2HG pool size is cooperatively regulated by both mitochondrial metabolism and glycolytic flux.

## DISCUSSION

### L-2HG accumulation is coordinately regulated by glycolysis and the TCA cycle

**Fig. 5.**
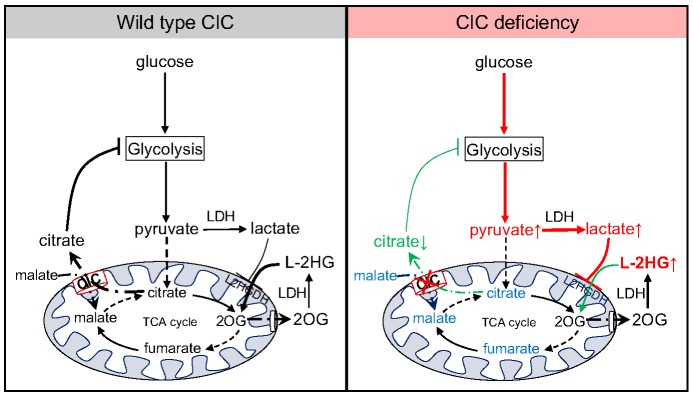
**Schematic of how the CIC influences L-2HG accumulation.** In wild-type larvae, CIC exports citrate from the mitochondria into the cytosol, which dampens glycolytic flux and restricts the amount of lactate produced by glycolysis. In contrast, mutations in *sea* result in decreased CIC activity, decreased cytosolic citrate levels and increased glycolytic flux. As a result, *sea* mutants synthesize excess lactate, which interferes with L2HGDH activity and promotes L-2HG accumulation. CIC, mitochondrial citrate carrier; LDH, lactate dehydrogenase; L-2HG, L-2-hydroxyglutarate; TCA, tricarboxylic acid; 2OG, 2-oxoglutarate.

The CIC plays a central role in cellular metabolism by controlling the amount of citrate that exits the TCA cycle and enters the cytosol. This function serves many purposes in cellular physiology, such as providing substrate for fatty acid synthesis, controlling histone acetylation and regulating cellular redox balance (Palmieri, 2013; Dolce et al., 2014; Morciano et al., 2009). In addition, cytosolic citrate serves to inhibit glycolytic flux, whether by inhibiting PFK protein in mammals (Kemp and Gunasekera, 2002), or by a yet to be determined mechanism in insects. This feedback mechanism fine-tunes central carbon metabolism by serving as a signal to slow glycolysis during times of sufficient energy production (Fig. 5). Our findings suggest that the role of citrate as a negative regulator of glycolysis represents the primary mechanism that induces L-2HG accumulation in *sea* mutants.

Based on our study and previous observations in human cell culture (Jiang et al., 2017), CIC deficiency induces elevated glycolytic flux and decreased citrate production (Fig. 5). As observed in *Drosophila*, human patients and CIC-deficient cells, these metabolic disruptions result in enhanced lactate synthesis, which, at least in the fly, inhibits dL2HGDH and stabilizes the L-2HG pool (Li et al., 2017; Jiang et al., 2017; Nota et al., 2013; Prasun et al., 2015). Such a model would also explain why citrate treatment could reduce 2HG levels in a patient with combined D-/L-2HGA (Muhlhausen et al., 2014), as partial restoration of cytosolic citrate levels would inhibit PFK enzyme activity and reduce glycolytic flux (Kemp and Gunasekera, 2002). Although it is tempting to speculate that citrate also inhibits *Drosophila* PFK, we would note that insect PFK homologs, when isolated from adult muscle tissue, are not inhibited by citrate (Nunes et al., 2016). Regardless of the mechanism, our findings demonstrate that citrate governs glycolytic flux in *Drosophila* larvae.

Our findings also highlight the role of dL2HGDH in regulating L-2HG accumulation. Although dLDH synthesizes most of the larval L-2HG pool, the kinetics of this reaction are poor and the only reasonable explanation for how flies accumulate such high L-2HG levels rests upon our observations that dL2HGDH activity is sensitive to lactate (Li et al., 2017). Because larval metabolism is highly glycolytic, aerobic lactate production stabilizes the L-2HG pool and allows for dramatic accumulation of this metabolite. Moreover, the positive correlation between L-2HG and lactate has been repeatedly observed in mammalian tissues such as mouse CD8^+^ T cells, human cells with disrupted 2OG metabolism and cell lines subjected to hypoxic conditions (Tyrakis et al., 2016; Burr et al., 2016; Intlekofer et al., 2015; Oldham et al., 2015). In this regard, we would also highlight the fact that mouse L2HGDH activity is inhibited by acidic pH (Nadtochiy et al., 2016), suggesting that even if lactate does not directly regulate L-2HG degradation, excess lactate accumulation could establish a microenvironment that stabilizes the L-2HG pool. Finally, our studies in the fly support a previous study of renal cell carcinomas, which revealed that aberrant L-2HG accumulation results from decreased L2HGDH activity (Shim et al., 2014). When considered in these contexts, our findings hint at a conserved feed-forward loop that links lactate synthesis with L-2HG accumulation, and suggest that an LDH inhibitor, such as oxamate, might alleviate the symptoms of combined D-/L-2HGA patients.

These results also raise the question of why D-2HG levels remain low in *sea* mutants. After all, D-2HG levels exceed those of L-2HG in both combined D-/L-2HGA patients and CIC-deficient cells. Although an adequate explanation requires a more detailed examination of *Drosophila* D-2HG metabolism, this discrepancy highlights a key difference between fly and human metabolism. Throughout our analyses, we repeatedly observed that flies accumulate minimal amounts of D-2HG, suggesting that the metabolic enzymes driving D-2HG accumulation in humans have either diverged in flies such that they no longer synthesize this molecule, or that D-2HG is only produced under specific cellular conditions. When considered in this context, the lack of *Drosophila* D-2HG production highlights the importance of L-2HG in cellular metabolism, as the mechanisms that control L-2HG accumulation are conserved across phyla. Moreover, these observations reinforce the notion that *Drosophila* genetics provides a powerful tool for dissecting the metabolic mechanisms that underlie L-2HG metabolism.

Finally, the *sea* mutant phenotypes raise important questions regarding the subcellular regulation of L-2HG metabolism. LDH-dependent production of L-2HG likely requires export of 2OG from the mitochondria into the cytosol. L2HGDH, however, is a mitochondrial enzyme (Rzem et al., 2004), thus raising the question of how L-2HG is transported across the mitochondrial membrane. Moreover, our results indicate that lactate is a key regulator of L2HGDH. Although the exact nature of this feedback mechanism remains to be elucidated, including how changes in CIC activity influences the activity, abundance and localization of the enzymes involved in lactate and L-2HG metabolism, our findings hint at the possibility that lactate is either synthesized in, or transported into, the mitochondria. Such a metabolic mechanism would be of significant interest considering recent excitement surrounding the proposed mitochondrial lactate shuttle (Brooks, 2018). Overall, these basic questions illustrate how little is known about the endogenous L-2HG metabolism and highlight the need for further study of this compound in model systems.

### The many faceted roles of 2HG in disease and metabolism

Combined D-/L-2HGA is associated with severe neurometabolic symptoms, developmental delays and childhood lethality (Muntau et al., 2000). Similarly, *sea* null mutations induce an early larval lethal phenotype, while the hypomorphic *sea* allele used in this study results in pupal lethality (Morciano et al., 2009). In addition, all examined *sea* mutant strains exhibit chromosomal abnormalities that include chromosome breaks and decreased histone acetylation (Morciano et al., 2009). Although these phenotypic parallels between flies and humans might suggest that 2HG accumulation is driving the *sea* mutant phenotypes, our ongoing studies indicate that larval development is largely resistant to elevated 2HG levels (Li et al., 2017). In fact, the amount of L-2HG in wild-type larvae is comparable to levels observed in L-2HGA and combined D-/L-2HGA patients (Muntau et al., 2000; Li et al., 2017; Barth et al., 1992), indicating that L-2HG, which is the primary 2HG enantiomer present in *sea* mutants, is not toxic to the development of flies maintained under standard culture conditions. Moreover, *dL2HGDH* mutant strains, which accumulate L-2HG to levels comparable with those seen in *sea* mutants, are completely viable and can be maintained as homozygous stocks (Li et al., 2017). These observations suggest that the *sea* mutant phenotypes potentially result from changes in cytosolic acetyl-CoA availability and altered redox balance. Although this result differs from humans, in whom the accumulation of L-2HG causes severe neurometabolic symptoms (Barth et al., 1992; Ye et al., 2018), it does not diminish the importance of studying *Drosophila* L-2HG metabolism because the metabolic mechanisms underlying L-2HG accumulation are conserved between flies and humans. Moreover, the ability of *Drosophila* to withstand high levels of L-2HG accumulation establishes the fly as an important model for studying combined D-/L-2HGA, as it provides a system in which to identify disease phenotypes that are caused by loss of CIC activity, but are not dependent on 2HG accumulation. Finally, our observations raise the question of why humans are sensitive to elevated L-2HG levels whereas flies are resistant. These organism-specific differences in L-2HG toxicity suggest that future comparisons of L-2HG metabolism in insects and mammals hold potential to identify factors that render cells sensitive to the pathological effects of this oncometabolite.

## MATERIALS AND METHODS

### *Drosophila* husbandry and genetics

Fly stocks were maintained on standard Bloomington *Drosophila* Stock Center (BDSC) media. Larvae were raised on molasses agar plates with yeast paste spread on the surface. All BDSC stocks used in this study are listed in Table S3. The *sea*^Δ*24*^ mutants and the precise excision control strain (*sea^Prec^*; previously noted as *Rev^24^*) were kindly provided by Dr Giovanni Cenci (Morciano et al., 2009). All experiments used a trans-heterozygous combination of *sea*^Δ*24*^ and a molecularly defined deficiency. All controls consisted of a trans-heterozygous combination of *sea^Prec^* and the same deficiency. The *UAS*-*sea* strain was generated by injecting the *Drosophila* Genomics Resource Center (DGRC) plasmid UFO06122 into BDSC stock 8621. *dL2HGDH* mutant strains used were as previously described (Li et al., 2017).

### Sample collection

Trans-heterozygous larvae of the appropriate genotypes (*sea*^Δ*24*^*/Df* and *sea^Prec^/Df*) were separated from siblings that harbored a *TM3, P{Dfd-GMR-nvYFP}3, Sb[1]* balancer chromosome based on the absence of YFP expression. For each experiment, individual samples were collected from independent mating bottles as described (Li and Tennessen, 2018). Each sample contained 15 middle-third-instar (mid-L3) larvae of mixed sex that were collected ∼12-24 h after the L2-L3 molt (∼84-96 h after egg laying). Larval samples were collected in 1.5 ml microfuge tubes, rinsed three times with ice-cold 0.9% NaCl and frozen in liquid nitrogen.

### Metabolomics and metabolic flux analysis

Samples for GC-MS analysis were processed as previously described (Li and Tennessen, 2018). For each experiment, six individual samples were collected from independent mating vials. All GC-MS experiments were repeated a minimum of two times. Metabolite extraction, derivatization and GC-MS analysis were conducted as described previously (Li et al., 2017; Li and Tennessen, 2018). Spectral data preprocessing was performed using MetAlign software (Lommen, 2009). Data were normalized to both the sample mass and a 2 µg/ml d4-succinic acid internal standard. For metabolic flux measurements, mid-L3 larvae were fed with semi-defined medium (Backhaus et al., 1984) containing 50% D-glucose-^13^C_6_ for 2 h, then metabolites were detected using GC-MS. Metabolites were detected using GC-MS and the isotopolog distributions were corrected based on the natural abundance of elements. All experiments were conducted a minimum of two times. The metabolic flux *f_x_* was estimated based on the formula X^L^/X^T^=p(1–exp(-f_x_*t/X^T^)), where *X^L^* is the amount of ^13^C-labeled metabolite, *X^T^* is the amount of total metabolite pool and *p* is the percentage of glucose-^13^C_6_.

### qRT-PCR

Total RNAs were extracted using Trizol reagent (ThermoFisher Scientific). cDNA was made using the Maxima First Strand cDNA Synthesis Kit (ThermoFisher Scientific), and quantitative PCR was performed using FastStart Essential DNA Green Master Kit (Roche Diagnostics) in a LightCycler 96 instrument (Roche Diagnostics). The primers for *rp49* (also known as *RpL32*) and *dLdh* were the same as reported previously (Li et al., 2017). Additional primer sequences are described in Table S4. mRNA levels were normalized to *rp49*.

### Statistical analysis

Multivariate data analysis (principal component analysis, PCA) was performed using MetaboAnalyst (Xia and Wishart, 2016). Statistical analysis was performed using GraphPad Prism 7.0c. Unless noted, two-tailed Student's *t*-test was used for univariate statistical analysis and Bonferroni correction was used for multiple comparisons.

### RNA-seq analysis

RNA was purified from staged mid-L3 larvae using an RNeasy Mini Kit (Qiagen). Sequencing was performed using an Illumina NextSeq500 platform with 75 bp sequencing module generating 41 bp paired-end reads. After the sequencing run, demultiplexing was performed with bcl2fastq v2.20.0.422. All files were trimmed using cutadapt v1.12 with the parameters: ‘-a AGATCGGAAGAGC -m 30 -q 30’ (Martin, 2011). Remaining read pairs were mapped against FlyBase 6.19 using hisat2 v2.1.0 with ‘–no-unal –no-mixed’ parameters (Kim et al., 2015). Gene counts were produced using HTseq-Count v0.9.0 (Anders et al., 2015). The R package DESeq2 was used to identify the differentially expressed genes (Love et al., 2014). The NCBI Gene Expression Omnibus (GEO) accession number for the RNA-seq data reported in this paper is GSE117117.

## Supplementary Material

Supplementary information

First Person interview
