# Supplementary material for: A Drosophila model of combined D-2- and L-2-hydroxyglutaric aciduria reveals a mechanism linking mitochondrial citrate export with oncometabolite accumulation
Source: Dis Model Mech. 2018 Sep 21;11(9):dmm035337. doi: 10.1242/dmm.035337 (PMC6177012; doi:10.1242/dmm.035337)
Supplement: Supplementary information [file dmm-11-035337-s1.pdf]

FIRST PERSON

First person – Hongde Li

First Person is a series of interviews with the first authors of a selection of papers published in Disease Models & Mechanisms, helping early-career researchers promote themselves alongside their papers. Hongde Li is first author on 'A *Drosophila* model of combined D-2- and L-2-hydroxyglutaric aciduria reveals a mechanism linking mitochondrial citrate export with oncometabolite accumulation', published in DMM. Hongde is a postdoctoral researcher in the lab of Jason Tennessen at Indiana University, Bloomington, USA, using genetic and metabolomic approaches to study biological functions of the oncometabolite L-2-hydoxyglutarate (2-HG). Specifically, he is interested in using *Drosophila melanogaster* to understand how L-2-HG metabolism is regulated *in vivo*.

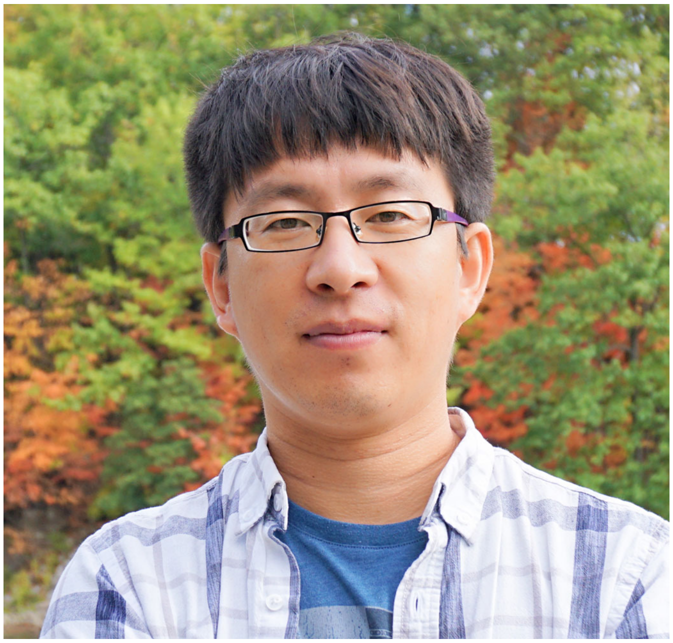

Hongde Li

How would you explain the main findings of your paper to non-scientific family and friends?

Human mutations in a gene encoding the protein mitochondrial citrate carrier (CIC) cause a rare inborn error of metabolism known as combined D-/L-2-hydroxyglutaric aciduria. A key feature of this disease is elevated levels of another protein, 2-hydroxyglutarate (2-HG), which are associated with severe neurological defects and developmental delay. In our study, we demonstrated that, in fruit flies, mutations in this gene induce metabolic defects that are similar to those observed in human patients. Moreover, we found that CIC regulates L-2-HG stability by governing the production of lactate, hinting at a potential treatment for patients with this disease.

What are the potential implications of these results for your field of research?

Prior to our work, the mechanism by which the loss of CIC function results in 2-HG accumulation was unknown. Our study reveals a previously unknown metabolic mechanism that explains why human CIC mutations result in elevated L-2-HG accumulation and provides a potential treatment strategy for combined D-/L-2HG aciduria.

What are the main advantages and drawbacks of the model system you have used as it relates to the disease you are investigating?

> "[T]he combination of a vast range of available genetic tools with modern metabolomic techniques […] can help to dissect the mechanisms underlying metabolic diseases in further detail."

There are many advantages to using the *Drosophila* model system. For my study, I think the most important one is the combination of a vast range of available genetic tools with modern metabolomic techniques, which can help to dissect the mechanisms underlying metabolic diseases in further detail. The drawback of using a *Drosophila* model to study D-/L-2-HG aciduria is that, unlike humans, the fly can withstand high levels of 2-HG without severe neurological defects. Regardless, the metabolic regulation of 2-HG accumulation is conserved between humans and flies.

Describe what you think is the most significant challenge impacting your research at this time and how will this be addressed over the next 10 years?

A significant challenge impacting our research is to study the metabolism of 2-HG in a tissue-specific manner at high resolution, because fruit flies are relatively small. With the development of modern mass spectrometry imaging techniques, this challenge will probably be addressed over the next 10 years.

What changes do you think could improve the professional lives of early-career scientists?

I think, for early-career scientists, persistence in research is very important. In addition, communication skills, hard work, and efficient time management are also essential.

What's next for you?

I will continue to use the genetic power of *Drosophila* models and metabolomics to study gene functions in the regulation of metabolic homeostasis.

Hongde Li's contact details: Department of Biology, Indiana University, 1001 East Third Street, Bloomington, IN 47405, USA.
E-mail: hl51@indiana.edu

Reference

Li, H., Hurlburt, A. J. and Tennessen, J. M. (2018). A *Drosophila* model of combined D-2- and L-2-hydroxyglutaric aciduria reveals a mechanism linking mitochondrial citrate export with oncometabolite accumulation. *Dis. Model. Mech.* **11**: dmm035337.

Disease Models & Mechanisms
